# Oral neonatal vitamin K deficiency bleeding prophylaxis in Switzerland (2018–2024), still valid guidelines for healthy infants

**DOI:** 10.1007/s00431-025-06730-4

**Published:** 2026-01-16

**Authors:** Bernard Laubscher, Gregor Schubiger, Mattia Rizzi

**Affiliations:** 1 Faculty of Biology and Medicine, University of Lausanne, Lausanne, Switzerland; 2 Division of Pediatrics, Department Women-Mother-Child, Lausanne University Hospital (CHUV), Rue du Bugnon 50, CH-1011 Lausanne, Switzerland; 3 Department of Pediatrics, Réseau Hospitalier Neuchâtelois, Neuchâtel, Switzerland; 4 Rischstrasse 3, Ebikon, Switzerland; 5 Pediatric Hematology-Oncology Unit, Division of Pediatrics, Department Woman-Mother-Child, Lausanne University Hospital (CHUV), Lausanne, Switzerland; 6 Istituto Pediatrico Della Svizzera Italiana (IPSI), Ente Ospedaliero Cantonale E Università Della Svizzera Italiana (USI), Servizio d’Emato-Oncologia Pediatrica, Bellinzona, Svizzera Switzerland

**Keywords:** Vitamin K deficiency bleeding, Vitamin K, Prophylaxis, Bleeding, Newborn, Infant

## Abstract

Vitamin K (VK) deficiency is a condition that puts newborn infants at increased risk of vitamin K deficiency bleeding (VKDB) during the first 6 months of life. In Switzerland, current prophylactic guidelines have been shown to prevent VKDB effectively in healthy infants. They were implemented in 2003 and prescribe oral administration of VK (2 mg oral Konakion® MM (mixt micellar) hour 4, day 4, and week 4). As prophylaxis parental refusal has been increasingly reported, we prospectively tested the Swiss VKDB prophylaxis validity using a nationwide surveillance program (Swiss Paediatric Surveillance Unit) designed to detect all hospitalized VKDB cases. During 6 years (September 1, 2018, until August 31, 2024), nine VKDB cases were reported for 505,708 live births (1.78/10^5^, 95% CI 0.81/10^5^–3.38/10^5^). All infants were exclusively breast fed and had VKDB risk factors such as parental VK refusal and/or cholestasis. Both Swiss VKDB 2018–2024 and 2005–2011 incidences were similar.

*Conclusion*: The Swiss VKDB prophylactic guidelines are still valid and effective for healthy infants if applied appropriately.

**Table:** 

**What is Known:** *• Infantile vitamin K deficiency bleeding (VKDB) can be prevented by either intramuscular or oral VK administration in healthy infants.* *• Parental VK prophylaxis refusal (reported as increasing in frequency) as well as unrecognized cholestasis are main risk factors for VKDB.*	
**What is New:** *• Switzerland’s 2003 VKDB prophylactic regimen (2 mg oral Konakion® MM (hour 4, day 4, and week 4) remains a valid recommendation for healthy infants.* *• Again, parental VK prophylaxis refusal and/or unrecognized cholestasis remain risk factors for VKDB.*

**What is Known:**

*• Infantile vitamin K deficiency bleeding (VKDB) can be prevented by either intramuscular or oral VK administration in healthy infants.*

*• Parental VK prophylaxis refusal (reported as increasing in frequency) as well as unrecognized cholestasis are main risk factors for VKDB.*

**What is New:**

*• Switzerland’s 2003 VKDB prophylactic regimen (2 mg oral Konakion® MM (hour 4, day 4, and week 4) remains a valid recommendation for healthy infants.*

*• Again, parental VK prophylaxis refusal and/or unrecognized cholestasis remain risk factors for VKDB.*

## Introduction

Vitamin K (VK) deficiency is a condition that puts newborn infants at increased risk of bleeding—a vitamin K deficiency bleeding (VKDB)—during the first 6 months of life [1]. VKDB can be prevented by a single intramuscular (IM) injection of VK at birth—regularly cited as the prophylaxis gold standard—although repeated oral VK doses have an almost similar efficacy in healthy infants [1, 2]. Worlwide, parental VK prophylaxis refusal, deemed by them as unnatural, unnecessary, or because of their broader stance against immunizations/injections, has been increasingly identified as a major risk for VKDB [2–8]. Parental IM (as opposed to oral) VK prophylaxis refusal puts all infants at risks of VKDB, perhaps even more so infants with unrecognized cholestasis. In Switzerland, current guidelines date from 2003 (2 mg oral Konakion® MM (mixt micellar) hour 4, day 4, and week 4, individual doses given by midwives (hour/day 4) and pediatricians (week 4, 1 month visit) and have been shown to be effective in healthy infants [2]. As societal acceptation of medical guidelines may change over time, we repeated a countrywide surveillance study to detect if Swiss prophylaxis to prevent VKDB was still efficient.

## Material and methods

The methodology used by the Swiss Paediatric Surveillance Unit (SPSU) has been described previously [9] and was used again for the present study. Using internationally accepted definitions, VKDB were classified as early, classical, or late VKDB and were either confirmed or probable (for the latter, in clinical practice, generally no proof of prothrombin time return to normal after VK administration since iv fresh plasma or coagulation factors are additionally often infused) [5, 10]. Data were prospectively collected from September 1, 2018, until August 31, 2024. The present report summarizes our 6-year experience and compares the incidence of VKDB with Swiss historical controls from the 2005 to 2011 study [2]. There were 505,708 live births in Switzerland during the study period [11]. The study was approved by our local Institutional Review Board (Commission cantonale d’éthique de la recherche sur l’être humain, CER-VD, 2018–00778).

### Statistical analysis

Where applicable, values are reported as median (minimum, maximum) or incidence with 95% confidence intervals (CI). The CI were computed according to Clopper-Pearson [12]. Chi square test with Yates correction for continuity [13] was used for comparison of different study populations.

## Results

The response rate of the surveyed units (*n* = 29) was 100%. Eleven cases were reported, two of which did not meet the case definition. Table 1 shows patients characteristics. Of the nine included cases, seven were definite and two probable. All were categorized as late VKDB with bleeding reported at a median age of 37 days (range 12–64 days). The overall definite and probable VKDB incidence was 9/505,708 (1.78/10^5^, 95% 0.81/10^5^–3.38/10^5^).

**Table 1:** 2018–2024 vitamin K deficiency bleeding cases in Switzerland

Case n°	Bleeding type	Vitamin K prophylaxis	Breast fed	Age at 1 st bleeding	Localization of bleeding	Hepatobiliary disease	Outcome
1	Definite Late	Full	Full	64 days	Intracranial	Biliary atresia	NA
2	Definite Late	Parents refusal	Full	43 days	Skin, intracranial	Cholestasis of unknown origin	NA
3	Definite Late	Parents refusal (daily oral phytomenadion, unknown quantity in mcg range)	Full	46 days	Skin, post testicular surgery	Cholestasis of unknown origin	Good
4	Probable Late	Full (1st 2 doses)	Full	19 days	Nose, skin, umbilical, intracranial	Cholestasis of unknown origin	Severe neurological handicap
5	Definite Late	Full	Full	44 days	Skin, nose, stool	Cholestatic giant cell hepatitis with undifferentiated paravertebral neuroblastoma	NA
6	Definite Late	Parents refusal	Full	33 days	Skin, subgaleal, intracranial	None	NA
7	Definite Late	Parents refusal	Full	23 days	Skin, intracranial	None	NA
8	Definite Late	Parents refusal	Full	37 days	Intracranial	None	NA
9	Probable Late	Parents refusal	Full	12 days	Umbilical	None	NA

*NA* not available

The bleeding was reported to be located, either alone or in combination, mostly in the skin (six cases) and intracranial (six cases, 1.19/10^5^, 95% CI 0.44/10^5^–2.58/10^5^), and less frequently in the umbilical area (three cases), the nose (two cases), the stool (one case), and following a surgical intervention (one case).

All infants were breast fed. Six (case 2, 3, 6, 7, 8, and 9) out of the nine included infants did not receive the recommended oral VK prophylaxis due to parental refusal. All three infants who received appropriate VK prophylaxis (case 1, 4, and 5) had cholestasis. One case (case 4) was not properly followed up initially and did not receive an adequate VK substitution after cholestasis detection. Five (case 1–5) out of the nine included infants had cholestasis, four of whom (case 1–3, 5) were diagnosed with cholestasis at the time of bleeding. Four (case 6, 7, 8, and 9) out of the nine included infants did not have cholestasis and had not received the recommended prophylaxis.

If patients who had received no or unrecommended VK prophylaxis were excluded (six cases), the incidence of late VKDB was 3/505,708 (0.59/105, 95% CI 0.12–1.73). If patients with cholestatic liver disease and inappropriate VK prophylaxis were excluded (nine cases), the incidence of late VKDB was 0/505,708 (0.00/105, 95% CI 0.00–0.73, no case).

As both 2005–2011 [2] and current studies share the same methodology within a constant VKDB prophylaxis environment (guidelines and costs coverage), we compared 2005–2011 and current data: there were no significant VKDB incidence changes between 2018–2024 and 2005–2011 (nine cases for 505,708 births versus five cases for 458,184 births [2]) (Chi square test with Yates correction for continuity, *P* = 0.5376). In total, both periods revealed 13 late VKDB cases (13/963,892 live births, 1.35/10^5^, 95% CI 0.72–2.31), 9/13 cases (7 definite, 2 probable) with cholestasis—of whom 2 with biliary atresia (BA) (1/2 with prophylaxis), 4 with parental VK refusal, and 1 with omission of the 3rd VK dose.

## Discussion

We have shown in the current surveillance study that VKDB incidence has remained stable in Switzerland in 2018–2024 compared to 2005–2011. Our data support, for the second time, the use of a VKDB prophylaxis with 2 mg oral Konakion® MM (hour 4, day 4, and week 4) for healthy infants.

Our findings keep in with other studies on all continents that have stressed that one major risk factor for VKDB was parental VK prophylaxis refusal [2–8]. Cholestasis, potentially resulting from congenital malformation (e.g., BA) or plurifactorial liver disease (such as giant cell hepatitis or alpha 1 antitrypsin deficiency or others), which often become clinically evident only after the neonatal period, is also a major VKDB risk factor.

Our data show that the Swiss oral 3 × 2 mg mixt micellar (hour 4, day 4, week 4) VKDB prophylaxis does not protect all apparently healthy neonates whose BA will become clinically evident only later or who will develop any other type of cholestasis in the first months of life.

In 2008, van Hasselt et al. concluded, in a retrospective Dutch and Danish analysis of BA, that a daily 25 ug oral VK dose failed to prevent bleedings in apparently healthy infants with unrecognized BA and that 1 mg weekly oral VK offered significantly higher protection to these infants and was of similar efficacy as 1 mg of IM prophylaxis at birth [14]. They underlined the fact that event analysis in specific populations at risk can help to evaluate and improve nationwide prophylactic regimens. In 2025, Verkade HJ shared his concerns about the apparently still very limited protection of the adapted Dutch oral VK prophylaxis (daily 150 ug oral VK) in infants with BA (personal communication HJ Verkade, Beatrix Children’s Hospital—University Medical Center Groningen, The Netherlands, and [15]). In Switzerland, between 2018 and 2024, 23 (23/505,708, 1:21,978) infants were diagnosed with a BA (personal communication B. Wildhaber, Swiss Pediatric Liver Center, Geneva, Switzerland) of whom one presented with an intracranial hemorrhage (case n°1, after full oral prophylaxis). Unfortunately, to date, no detailed information is available on whether the infants were breast or formula-fed, on the given prophylaxis or initial coagulation studies at diagnosis.

Failure of IM VK prophylaxis is certainly rare but has been reported in infant with *and* without cholestatic liver disease, fi by Zurinsky et al. (7/42 late VKDB cases after 1 mg VK IM) who also stressed that parental VK prophylaxis refusal was becoming more frequent [5]. In this regard, we might fear that a return to generalized IM prophylaxis could lead even more parents to refuse any VK, ultimately putting more infants (with or without cholestasis) at risk of VKDB.

Underreporting could be the main limitation of our study. Severe VKDB such as intracranial hemorrhages are however likely to be reported which seems to be supported by the concordance of intracranial VKDB cases independently reported to the SPSU and to the national unit for infantile BA. Benign VKDB, easily managed with oral VK by general pediatricians, could represent underreported prophylactic failures. They would however be underreported irrespective of the VK prophylactic regimen (oral versus IM) allowing valid inter-prophylactic regimen comparisons. We are further confident that the 2005–2011 and 2018–2024 questionnaires 100% response rates support valid data to evaluate the efficacy of the Swiss VKDB prophylaxis.

## Conclusion

Our current countrywide surveillance study:

Reinforces our 2005–2011 conclusions that the oral neonatal VKDB prophylaxis in Switzerland (2 mg oral Konakion® MM (hour 4, day 4, and week 4) remains valid and effective for healthy infants, if applied appropriately.

Stresses that parental VK prophylaxis refusal is the main risk factor for VKDB.

Emphasizes that the Swiss VKDB prophylaxis does not completely protect cholestatic infants from late VKDB.

Should promote retrospective and prospective studies of all BA cases in countries applying the 3 × 2 mg VK prophylaxis (Switzerland and Germany) with regard to VKDB.

Supports Sirachainan et al.’s conclusions in a recent multinational study covering 38 countries [6], that an optimal VKDB prophylaxis for infants with liver disease should be established, and that comprehensive education for parents and communities on VKDB complications is essential.

## Data Availability

No datasets were generated or analysed during the current study.

## Abbreviations

BA: Biliary atresia
CI: Confidence intervals
IM: Intramuscular
MM: Mixt micellar
SPSU: Swiss Paediatric Surveillance Unit
VK: Vitamin K
VKDB: Vitamin K deficiency bleeding
